# Association between Omega-3 Index and Hyperglycemia Depending on Body Mass Index among Adults in the United States

**DOI:** 10.3390/nu14204407

**Published:** 2022-10-20

**Authors:** Sunyoung Jo, William S. Harris, Nathan L. Tintle, Yongsoon Park

**Affiliations:** 1 Department of Food and Nutrition, Hanyang University, Seoul 04763, Korea; 2 National Evidence Based Healthcare Collaborating Agency, Seoul 04933, Korea; 3 Sanford School of Medicine, University of South Dakota, Sioux Falls, SD 57105, USA; 4 Fatty Acid Research Institute, Sioux Falls, SD 57106, USA; 5 Department of Population Health Nursing Science, College of Nursing, University of Illinois-Chicago, Chicago, IL 60612, USA

**Keywords:** body mass index, Omega-3 Index, *n*-3 polyunsaturated fatty acids, hyperglycemia, type 2 diabetes

## Abstract

There is inconsistency regarding the association between long-chain *n*-3 polyunsaturated fatty acids such as eicosapentaenoic acid (EPA; 20:5n3) and docosahexaenoic acid (DHA; 22:6n3) and the risk of type 2 diabetes. The present study aimed to investigate the association between the Omega-3 Index (erythrocyte EPA + DHA) and glycemic status as a function of body mass index (BMI). Cross-sectional data from routine clinical laboratory testing with a total of 100,572 people aged over 18 years and BMI ≥ 18.5 kg/m^2^ were included. Of the patients, 10% were hyperglycemic (fasting plasma glucose levels ≥ 126 mg/dL) and 24.7% were of normal weight, 35.0% were overweight, and 40.3% were obese. Odds ratios (ORs) of being hyperglycemic were inversely associated with the Omega-3 Index, but weakened as BMI increased. Thus, ORs (95% CI) comparing quintile 5 with quintile 1 were 0.54 (0.44–0.66) in the normal weight group, 0.70 (0.61–0.79) in the overweight group, and 0.74 (0.67–0.81) in the obese group. Similar patterns were seen for EPA and DHA separately. The present study suggested that a low Omega-3 Index is associated with a greater risk of disordered glucose metabolism and this is independent of BMI.

## 1. Introduction

Type 2 diabetes (T2D) is characterized by a chronic state of hyperglycemia due to relative insulin deficiency and insulin resistance [1]. Obesity is a major risk factor for T2D by increasing insulin resistance [2], and 89% of patients with T2D are overweight or obese in the United States (USA) [3]. Diet is an important modifiable factor to prevent and manage T2D [4]. The American Diabetes Association recommends eating fatty fish rich in long-chain *n*-3 polyunsaturated fatty acids (PUFAs) including eicosapentaenoic acid (EPA; 20:5n3) and docosahexaenoic acid (DHA; 22:6n3), and seeds rich in α-linolenic acid (ALA; 18:3n3) to prevent or treat cardiovascular disease in patients with diabetes [5]. However, the dietary supplementation of *n*-3 PUFAs is not recommended to improve glycemic management [5,6,7]. Although some meta-analyses of prospective cohort studies found that the risk of T2D was not associated with intake [8] and blood levels of *n*-3 PUFAs [9], an analysis of UK Biobank data showed a lower risk for T2D in participants reporting fish oil use and eating ≥2 servings/week of oily fish than <1 serving/week [10]. In addition, a pooled analysis of prospective cohort studies reported that the incidence of T2D was negatively associated with higher long-chain *n*-3 PUFAs, but not ALA, measured in adipose tissue or blood [11]. Consistently, previous studies showed that blood levels of long-chain *n*-3 PUFA were negatively correlated with hemoglobin A1c (HbA1c), levels of insulin, and homeostatic model assessment for insulin resistance (HOMA-IR) in Australian, Chinese, Korean, New Zealand, and Taiwanese adults with or without T2D, suggesting that long-chain *n*-3 PUFA could have a beneficial role in the risk of T2D [12,13,14,15,16]. In a diabetic rodent model, the supplementation of long-chain *n*-3 PUFAs also improved insulin resistance and glucose tolerance by increasing hepatic insulin sensitivity [17,18].

Previous meta- or pooled analyses of prospective cohort studies included various long-chain *n*-3 PUFA biomarkers measured in adipose tissue, plasma, serum, and erythrocyte [9,11]. The Omega-3 Index, the sum of EPA + DHA in relation to total fatty acid content in red blood cell membranes, has been suggested to be superior to plasma or serum long-chain *n*-3 PUFAs because of its reflection of long-term dietary intake of long-chain *n*-3 PUFA [19]. In addition, the Omega-3 Index has been known for the association with various factors including age, sex, education, waist circumference, smoking, and genotype [20,21], and especially negative correlation with body mass index (BMI), a major risk factor for T2D [22,23]. Qian et al. [11] suggested the inverse association between long-chain *n*-3 PUFAs and the incidence of T2D was stronger in obese than non-obese participants. 

To the best of our knowledge, there is no study evaluating the association between the Omega-3 Index and hyperglycemia by BMI categories. Therefore, the present study tested the hypothesis that the Omega-3 Index is negatively associated with hyperglycemia and is inversely correlated with markers of glucose metabolism including HbA1c, levels of insulin, and HOMA-IR. 

## 2. Materials and Methods

### 2.1. Participants

This cross-sectional study was based on routine clinical laboratory data from Health Diagnostic Laboratory, Inc. (HDL, Inc., Richmond, VA, USA) between 2011 and 2012. Among 100,572 patients 18+ years old and with BMI ≥ 18.5 kg/m^2^, 10,222 were considered to have hyperglycemia (fasting plasma glucose levels ≥ 126 mg/dL). Patients were classified as normal weight (BMI: 18.5–24.9 kg/m^2^), overweight (BMI: 25.0–29.9 kg/m^2^), or obese (BMI: ≥30.0 kg/m^2^). Underweight (BMI: <18.5 kg/m^2^) patients were excluded. The study was conducted in accordance with the Declaration of Helsinki, and a waiver of informed consent requirements for this study (which used only deidentified and aggregated laboratory data) was obtained from the University of South Dakota (IRB-21-147). 

### 2.2. Laboratory Methods

Overnight fasting blood samples were collected at clinics across the USA and shipped with cold packs to HDL, Inc. for biochemical measurements. Plasma levels of glucose and serum levels of insulin were measured using an automated analyzer. HbA1c was measured using high-performance liquid chromatography. HOMA-IR was calculated as fasting insulin (μU/mL) × fasting glucose (mg/dL)/405 [24]. Erythrocyte *n*-3 PUFA composition was measured as described previously [25]. Fatty acid methyl esters were generated by treatment with boron trifluoride methanol benzene (10 min at 100 °C) (Sigma-Aldrich, St. Louis, MO, USA), extracted with water and hexane and analyzed by gas chromatography (Shimadzu 2010AF; Shimadzu Scientific Instrument, Kyoto, Japan), equipped with a 100 m × 0.25 mm inner diameter, with a 0.20 μm film capillary column (SP2560; Supelco, Bellefonte, PA, USA). Using standard mixture (GLC-727; Nu-Check Prep, Elysian, MN, USA), individual fatty acids were identified and expressed as a percentage of the total identified fatty acids. Every batch was quantified by measuring the coefficient of variation of the Omega-3 Index in quality control sample.

### 2.3. Statistical Methods

Differences between hyperglycemic and normoglycemic patients were assessed using Student’s *t* tests for continuous variables reported as means ± standard deviation, and Chi-squared tests for proportions of dichotomous variables. Correlation between erythrocyte *n*-3 PUFA composition, and HbA1, levels of insulin, and HOMA-IR was evaluated using Pearson’s correlation coefficient after adjusting for age, sex, and BMI. Multivariable logistic regression analysis was applied to estimate the ORs of being hyperglycemic with quintiles of erythrocyte *n*-3 PUFA composition by using lowest quintiles set as the reference group (OR = 1.0) after adjusting for age, sex, and BMI. In addition, linear trend analysis across quintiles was performed. The interactions between *n*-3 PUFA composition and weight groups on being hyperglycemic were tested using a two-way ANOVA. All statistical analyses were performed using SAS software, version 9.4 (SAS Institute, Cary, NC, USA). Values of *p* < 0.05 were considered to be statistically significant.

## 3. Results

Patient characteristics are shown in Table 1. The weight distributions in each BMI category were 24.7% normal weight, 35.0% overweight, and 40.3% obese. Hyperglycemic patients were significantly older, predominantly male, and heavier in all weight groups compared with normoglycemic patients. Levels of glucose and insulin, HbA1c, and HOMA-IR were significantly higher in hyperglycemic than normoglycemic patients in all weight groups. 

Erythrocyte levels of EPA was significantly lower in hyperglycemic than normoglycemic patients in all weight groups, and the Omega-3 Index and erythrocyte levels of DHA were significantly lower in hyperglycemic than normoglycemic patients in the normal weight group. Erythrocyte levels of ALA were significantly higher in hyperglycemic than normoglycemic patients in the obese group.

As shown in Table 2, there were significant interactions between quintiles of the Omega-3 Index (and erythrocyte levels of EPA and DHA) with weight group on hyperglycemic status. Within weight groups, the ORs of being hyperglycemic showed inverse associations with the Omega-3 Index and erythrocyte levels of EPA and DHA. However, the ORs for the overweight and obese groups were higher than those for the normal weight group, particularly in the highest quintile of the Omega-3 Index. The ORs of hyperglycemia showed inverse associations with erythrocyte levels of ALA in the overweight and obese groups, but not in the normal weight group. There were no interactions between quintiles of erythrocyte levels of ALA on hyperglycemic status.

HbA1c was negatively correlated with erythrocyte levels of ALA, EPA, DHA, and the Omega-3 Index among all weight groups (Table 3). Levels of insulin were negatively correlated with erythrocyte levels of EPA among all weight groups and with erythrocyte levels of DHA and the Omega-3 Index in the normal weight group, but positively correlated with erythrocyte levels of DHA in the obese group. HOMA-IR was negatively correlated with erythrocyte levels of EPA, but not DHA in all weight groups, and with the Omega-3 Index in the normal weight group. In addition, correlation between levels of insulin and HOMA-IR and erythrocyte levels of ALA was negative in the normal weight group, but positive in the overweight and obese groups.

## 4. Discussion

The present study showed that higher Omega-3 Index and erythrocyte levels of EPA and DHA were associated with lower odds of hyperglycemia, and the association was stronger in the normal weight than the obese group. In addition, HbA1c was negatively correlated with the Omega-3 Index and erythrocyte levels of EPA and DHA in all weight groups. Levels of insulin and HOMA-IR were negatively correlated with erythrocyte levels of EPA in all weight groups, and with the Omega-3 Index in the normal weight group, but not in the overweight and obese groups.

Previous studies reported that the Omega-3 Index was negatively correlated with HbA1, levels of insulin, and HOMA-IR [13,15], and inversely associated with the risk of T2D [11,14]. Moreover, dietary supplementation of EPA and DHA decreased the levels of glucose and HbA1c [26,27,28]. Long-chain *n*-3 PUFAs are an important component of phospholipids in cell membranes and can indirectly influence the expression of genes regulating glucose metabolisms and insulin signaling [29,30]. Pooled analysis of prospective cohort studies also reported negative association between the incidence of T2D and long-chain *n*-3 PUFAs measured in adipose tissue or blood [11], but the meta-analysis of published prospective cohort studies reported that the risk of T2D was not associated with blood levels of long-chain *n*-3 PUFAs [9]. First, this discrepancy could be partly due to the difference in study population. Chen et al. [9] included studies performed in Europe, Australia, and Asia, but Qian et al. [11] also included studies conducted in the USA. There were only two published studies in the US subjects, and in them, there was a lower risk of T2D associated with higher blood levels of EPA + DHA [31]. A study in elderly American women found the risk of T2D was inversely associated with the Omega-3 Index [32], which was consistent with our findings. The Omega-3 Index is the validated biomarker of tissue levels and intake of long-chain *n*-3 PUFAs, and originally developed as a risk factor for coronary heart disease, categorized as desirable (≥8%), suboptimal (<8 to 4%), and low (≤4%) levels [33]. Omega-3 Index has been reported to be lower in the USA (less than 4% to 5%) than in European countries (4% to more than 8%) and Australia (4–6%) [34,35]. The intake of long-chain *n*-3 PUFA in the USA (100–149 mg/day) was also lower than in European countries (150 to more than 550 mg/day) and Australia (250–349 mg/day) [36], suggesting different levels of the Omega-3 Index or long-chain *n*-3 PUFA intake might in part explain this inconsistency. In addition, Qian et al. [11] observed that the sum of long-chain *n*-3 PUFAs including EPA, docosapentaenoic acid (22:5n3; DPA), and DHA were negatively associated with the risk of T2D, but Chen et al. [9] did not report total long-chain *n*-3 PUFAs. The sum of all three long-chain *n*-3 PUFAs may be a better biomarker than each individual *n*-3 PUFA because there is some interconversion among long-chain *n*-3 PUFAs [37]. Djoussé et al. [31] also reported that the risk of incident of T2D was negatively associated with plasma total long-chain *n*-3 PUFAs, but not associated with individual EPA and DHA. 

Since most patients with T2D are obese, BMI is a major risk factor for the development of T2D [2]. The Omega-3 Index and blood levels of long-chain *n*-3 PUFAs were negatively correlated with BMI and glucose in previous cross-sectional studies [13,16,20,21,38]. Moreover, BMI may modulate the negative association of blood levels of long-chain *n*-3 PUFAs with the risk of T2D [39]. Qian et al. [11] reported the stronger negative association of long-chain *n*-3 PUFAs with the risk of T2D in participants with BMI over 30 kg/m^2^ than under (relative risk: 0.70, 0.86, respectively). Abbott et al. [39] also showed that plasma levels of EPA + DHA were negatively associated with the risk of T2D in women with BMI over 25 kg/m^2^, but not under 25 kg/m^2^. The present study observed that there was a similar inverse association in patients with BMI over 25 or 30 kg/m^2^, but stronger inverse association in patients with BMI under 25 or 30 kg/m^2^. In our multivariable regression analysis, the association of the Omega-3 Index with hyperglycemia was attenuated in the overweight and obese groups after additional adjustment for BMI, as compared with normal weight group (data not shown). The results suggested that BMI had a greater impact on the association between the Omega-3 Index and risk of hyperglycemia in the obese than normal weight group. In our multivariable regression analysis, the association of the Omega-3 Index with hyperglycemia were significant at quintile 3 in the overweight (*p* < 0.001) and obese (*p* < 0.001) groups but not in the normal weight group (*p* = 0.054). However, ORs at quintile 3 were not statistically significant in all weight groups (data not shown).

One possible explanation regarding the inconsistency between the present and previous studies may be the different biomarker compartment measured. The present study measured long-chain *n*-3 PUFA in erythrocyte, while Qian et al. [11] used adipose tissue, plasma, serum, or erythrocyte, and Abbott et al. [39] used plasma. Erythrocyte long-chain *n*-3 PUFA, especially Omega-3 Index has been suggested to be superior to plasma or serum long-chain *n*-3 PUFA since its reflection of long-term dietary intake of long-chain *n*-3 PUFA [19]. In addition, Qian et al. [11] did not examine association as a function of BMI categories, and Abbott et al. [39] had small numbers of normal weight participants with T2D.

Bhaswant et al. [40] showed that EPA and DHA were different in their effectiveness to improve insulin resistance and promote insulin secretion, since EPA, but not DHA was involved in activating G-protein-coupled receptor 40 and insulin-like growthfactor-1 pathway. In this study, levels of insulin and HOMA-IR were negatively correlated with erythrocyte levels of EPA, whereas not with erythrocyte levels of DHA. A meta-analysis reported insulin sensitivity was significantly improved in EPA-enriched group (1 ≤ ratio EPA to DHA), but not in DHA-enriched group (1 > ratio EPA to DHA) [41]. Interestingly, the present study also showed that erythrocyte levels of DHA were negatively correlated with insulin in the normal weight group, while positively in the obese group. Similarly, Iggman et al. [42] observed that adipose tissue levels of DHA were negatively correlated with insulin sensitivity in the overweight and obese groups, but not in underweight and normal weight groups. In addition, supplementation of DHA significantly increased insulin in overweight hyperlipidemic men [43]. The putative adverse effect of DHA on insulin in obese participants may be caused by the impact of hepatic insulin or insulin secretion rates through an increased hepatic glucose output [44,45]. 

Unlike long-chain *n*-3 PUFA, adipose-tissue or blood levels of ALA were not associated with the risk of T2D in a meta- and pooled analyses of prospective cohort studies [9,11]. The present study consistently observed no association of erythrocyte levels of ALA with the risk of T2D in underweight and normal weight groups, but positive association in the overweight and obese groups. Takkunen et al. [46] also observed serum levels of ALA were associated with higher incidence of T2D in overweight and obese participants. Moreover, Petersen et al. [16] found blood levels of ALA were positively associated with glucose, insulin, and HOMA-IR in participants whose BMI was similar to our participants. A meta-analysis of randomized controlled trials reported that the supplementation of ALA increased insulin levels in participants with or without T2D [6]. Therefore, the inconsistency of association between blood levels of ALA and the risk of T2D might be partly due to the weight status, since the majority of previous studies conducted with relatively low BMI. Although the mechanisms of how ALA has effect on T2D by weight status are unknown, ALA has been shown to enhance the development of pro-inflammatory environment within the vascular endothelium in vitro study [47], known as a marker of T2D [48].

This present study had a few limitations. First, although some potential confounders were adjusted for statistically, other factors affecting hyperglycemia and erythrocyte levels of long-chain *n*-3 PUFA may have played a role. Particularly, because of a lack of information on medical history, patients with gestational diabetes might be included, and BMI was self-reported. Second, since the USA is a country known to have a lower Omega-3 Index than Asian populations, the findings may not be generalized to other populations. Finally, because of the cross-sectional study design, only associations rather than cause–effect relationships between erythrocyte long-chain *n*-3 PUFA composition and hyperglycemia are defined.

In conclusion, the present study found that an inverse association exists between the Omega-3 Index and glycemic status, and that this relationship was modified by BMI. Although there was a suggestion that the relationship between the Omega-3 Index and glycemic status might be a little different across BMI, the interaction was not strong and likely to be of no clinical significance. Thus, regardless of BMI, the higher the Omega-3 Index, the lower the odds of being hyperglycemic. Further studies of this question in large, population-based longitudinal studies with homogeneous samples of diverse geographical regions are needed.

## Figures and Tables

**Table 1:** Characteristics and erythrocyte long-chain *n*-3 polyunsaturated fatty acid composition of patients (*n* = 100,572) ^1^.

	Normal Weight (*n* = 24,901)			Overweight (*n* = 35,175)			Obese (*n* = 40,496)		
	Hyperglycemic (*n* = 918)	Normoglycemic (*n* = 23,983)	*p*-Value	Hyperglycemic (*n* = 2698)	Normoglycemic (*n* = 32,477)	*p*-Value	Hyperglycemic (*n* = 6606)	Normoglycemic (*n* = 33,890)	*p*-Value
Age (y)	64.0 ± 14.4	54.5 ± 16.7	<0.001	62.9 ± 12.3	56.0 ± 14.7	<0.001	58.4 ± 11.8	53.7 ± 14.0	<0.001
Women (%)	46.1	68.0	<0.001	35.1	45.3	<0.001	42.4	52.6	<0.001
BMI (kg/m^2^)	23.0 ± 1.5	22.5 ± 1.7	<0.001	27.7 ± 1.4	27.4 ± 1.4	<0.001	37.1 ± 6.5	35.6 ± 5.5	<0.001
Glucose (mg/dL)	180.8 ± 69.3	88.8 ± 10.7	<0.001	175.8 ± 62.1	92.6 ± 11.2	<0.001	177.8 ± 57.1	95.1 ± 12.2	<0.001
HbA1c (%)	7.8 ± 2.2	5.3 ± 0.5	<0.001	7.7 ± 1.9	5.4 ± 0.5	<0.001	7.9 ± 1.8	5.6 ± 0.6	<0.001
Insulin (µU/mL)	14.9 ± 24.4	7.1 ± 6.9	<0.001	19.4 ± 26.9	10.5 ± 9.9	<0.001	27.1 ± 31.4	17.4 ± 17.4	<0.001
HOMA-IR	6.5 ± 12.8	1.6 ± 1.6	<0.001	8.3 ± 12.3	2.5 ± 2.7	<0.001	11.7 ± 14.8	4.2 ± 4.3	<0.001
ALA	0.14 ± 0.05	0.15 ± 0.05	0.158	0.140 ± 0.048	0.139 ± 0.043	0.141	0.137 ± 0.041	0.135 ± 0.038	0.024
EPA	0.72 ± 0.63	0.85 ± 0.75	<0.001	0.71 ± 0.60	0.76 ± 0.62	<0.001	0.61 ± 0.48	0.63 ± 0.49	<0.001
DHA	4.31 ± 1.66	4.50 ± 1.54	0.001	4.272 ± 1.484	4.269 ± 1.417	0.924	3.91 ± 1.29	3.92 ± 1.26	0.463
Omega-3 Index	5.03 ± 2.18	5.35 ± 2.14	<0.001	4.98 ± 1.97	5.03 ± 1.92	0.237	4.52 ± 1.68	4.56 ± 1.65	0.104

BMI, body mass index; HbA1c, hemoglobin A1c; HOMA-IR, homoeostatic model assessment for insulin resistance; ALA, α-linolenic acid; EPA, eicosapentaenoic acid; DHA, docosahexaenoic acid; Omega-3 Index, 20:5n3 + 22:6n3. ^1^ Values are mean ± standard deviation or %, as appropriate (Student’s *t* test); Hyperglycemic and normoglycemic were referred to as fasting glucose level ≥ 126 mg/dL and fasting glucose level < 126 mg/dL, respectively.

**Table 2:** Association between erythrocyte *n*-3 polyunsaturated fatty acids and hyperglycemia by multivariate regression analysis ^1^.

		Quintiles of Erythrocyte *n*-3 Polyunsaturated Fatty Acid Content					*p*-Value for Trend ^2^	*p*-Value for Interaction with Weight Groups ^3^
		1	2	3	4	5		
ALA	Cutoff (%)	≤0.10	0.10< to ≤0.12	0.12< to ≤0.14	0.14< to ≤0.17	>0.17		0.712
	Normal weight							
	Cases/controls (*n*)	175/4011	169/4169	179/4810	170/4964	225/6029		
	OR (95% CI)	1.00	1.05 (0.85–1.31)	1.07 (0.86–1.33)	1.01 (0.81–1.26)	1.15 (0.94–1.42)	0.275	
	Overweight							
	Cases/controls (*n*)	634/6863	502/6445	502/6748	496/6139	564/6282		
	OR (95% CI)	1.00	0.91 (0.81–1.03)	0.91 (0.81–1.03)	1.03 (0.91–1.16)	1.18 (1.05–1.33) ^4^	0.003	
	Obese							
	Cases/controls (*n*)	1569/7725	1308/6919	1322/7107	1200/6431	1207/5708		
	OR (95% CI)	1.00	0.99 (0.91–1.07)	1.04 (0.95–1.12)	1.09 (1.00–1.19) ^4^	1.32 (1.21–1.43) ^5^	<0.001	
EPA	Cutoff (%)	≤0.34	0.34< to ≤0.45	0.45< to ≤0.61	0.61< to ≤0.98	>0.98		0.010
	Normal weight							
	Cases/controls (*n*)	233/4478	194/3925	142/4073	158/4957	191/6550		
	OR (95% CI)	1.00	0.89 (0.73–1.09)	0.62 (0.50–0.77) ^4^	0.53 (0.43–0.66) ^4^	0.44 (0.36–0.54) ^4^	<0.001	
	Overweight							
	Cases/controls (*n*)	623/5793	555/6089	484/6488	494/7009	542/7098		
	OR (95% CI)	1.00	0.83 (0.74–0.94) ^5^	0.66 (0.59–0.75) ^4^	0.58 (0.51–0.66) ^4^	0.58 (0.51–0.65) ^4^	<0.001	
	Obese							
	Cases/controls (*n*)	1659/7388	1624/7832	1367/7449	1093/6370	863/4851		
	OR (95% CI)	1.00	0.90 (0.84–0.98) ^5^	0.79 (0.73–0.85) ^4^	0.69 (0.63–0.75) ^4^	0.65 (0.59–0.71) ^4^	<0.001	
DHA	Cutoff (%)	≤2.95	2.95< to ≤3.59	3.59< to ≤4.32	4.32< to ≤5.36	>5.36		
	Normal weight							0.010
	Cases/controls (*n*)	197/3959	168/3791	169/4312	160/5129	224/6792		
	OR (95% CI)	1.00	0.87 (0.70–1.08)	0.74 (0.60–0.92) ^5^	0.56 (0.45–0.70) ^4^	0.56 (0.46–0.69) ^4^	<0.001	
	Overweight							
	Cases/controls (*n*)	513/5944	529/6125	521/6351	557/6987	578/7070		
	OR (95% CI)	1.00	0.91 (0.80–1.04)	0.82 (0.72–0.93) ^5^	0.76 (0.67–0.86) ^4^	0.72 (0.64–0.82) ^4^	<0.001	
	Obese							
	Cases/controls (*n*)	1552/7975	1610/7891	1422/7316	1127/6158	895/4550		
	OR (95% CI)	1.00	0.97 (0.90–1.05)	0.89 (0.82–0.96) ^5^	0.79 (0.72–0.86) ^4^	0.80 (0.73–0.88) ^4^	<0.001	
Omega-3 Index	Cutoff (%)	≤3.35	3.35< to ≤4.04	4.04< to ≤4.93	4.93< to ≤6.33	>6.33		
	Normal weight							0.036
	Cases/controls (*n*)	192/3986	177/3796	176/4247	157/5138	216/6816		
	OR (95% CI)	1.00	0.92 (0.75–1.14)	0.81 (0.66–1.00)	0.57 (0.46–0.71) ^4^	0.54 (0.44–0.66) ^4^	<0.001	
	Overweight							
	Cases/controls (*n*)	525/5923	542/6043	516/6401	537/7011	578/7099		
	OR (95% CI)	1.00	0.93 (0.82–1.06)	0.79 (0.70–0.90) ^4^	0.71 (0.62–0.81) ^4^	0.70 (0.61–0.79) ^4^	<0.001	
	Obese							
	Cases/controls (*n*)	1596/7896	1608/7973	1438/7325	1101/6157	863/4539		
	OR (95% CI)	1.00	0.93 (0.86–1.00)	0.86 (0.80–0.94) ^4^	0.74 (0.68–0.80) ^4^	0.74 (0.67–0.81) ^4^	<0.001	

ALA, α-linolenic acid; EPA, eicosapentaenoic acid; DHA, docosahexaenoic acid; Omega-3 Index, 20:5n3 + 22:6n3. ^1^ Values are odds ratios (OR) with 95% confidence intervals (CI) after adjusting for age, sex, and BMI; Normal weight is if BMI is 18.5 to <25.0 kg/m^2^; Overweight is if BMI is 25.0 to <30.0 kg/m^2^; Obese is if BMI is ≥30.0 kg/m^2^. ^2^ The likelihood ratio test was used for the detection of linear trend. ^3^ *p*-value for the interaction with BMI was determined using a two-way ANOVA. ^4^ *p*-value of <0.001 compared with the first quintile by logistic regression analysis. ^5^ *p*-value of <0.05 compared with the first quintile by logistic regression analysis.

**Table 3:** Pearson’s correlation coefficient between erythrocyte long-chain *n*-3 polyunsaturated fatty acid composition and markers of glucose metabolism (*n* = 100,572) ^1^.

		ALA	*p*-Value	EPA	*p*-Value	DHA	*p*-Value	Omega-3 Index	*p*-Value
HbA1c	Normal weight (*n* = 24,901)	0.007	0.259	−0.066	<0.001	−0.059	<0.001	−0.066	<0.001
	Overweight (*n* = 35,175)	0.036	<0.001	−0.071	<0.001	−0.058	<0.001	−0.067	<0.001
	Obese (*n* = 40,496)	0.046	<0.001	−0.070	<0.001	−0.066	<0.001	−0.071	<0.001
Insulin	Normal weight (*n* = 24,901)	−0.023	<0.001	−0.044	<0.001	−0.013	0.043	−0.025	<0.001
	Overweight (*n* = 35,175)	0.014	0.009	−0.019	<0.001	0.007	0.164	−0.001	0.915
	Obese (*n* = 40,496)	0.016		−0.010	0.049	0.013	0.008	0.007	0.144
HOMA-IR	Normal weight (*n* = 24,901)	−0.014	0.014	−0.038	<0.001	−0.012	0.056	−0.022	<0.001
	Overweight (*n* = 35,175)	0.017	0.001	−0.024	<0.001	−0.001	0.892	−0.008	0.111
	Obese (*n* = 40,496)	0.028	<0.001	−0.015	0.004	−0.0004	0.936	−0.005	0.350

ALA, α-linolenic acid; EPA, eicosapentaenoic acid; DHA, docosahexaenoic acid; HbA1c, hemoglobin A1c; HOMA-IR, homoeostatic model assessment for insulin resistance; Omega-3 Index, 20:5n3 + 22:6n3. ^1^ Pearson’s correlation coefficient (r) was adjusted for age, sex, and BMI.
